# The Late Sir Henry Burdett

**Published:** 1920-05-22

**Authors:** 


### THE LATE SIR HENRY BURDETT.

Expressions of sympathy continue to reach us from many quarters. The Governors of St. Thomas's Hospital, at their meeting on the 6th instant, unanimously passed a vote of condolence with the family of the late Sir Henry Burdett. The Governors "deplore his loss as a Governor of the hospital, who took a deep interest in its affairs," and at the same time they express the feeling that the cause of the Voluntary Hospitals has "suffered deeply in the death of one of its strongest supporters."

At a meeting of the Committee of Management of the Corporation of the Dreadnought Seamen's Hospital, Greenwich, Sir Perceval Nairne, presiding over a very large attendance, the death of Sir Henry Burdett was announced. The Chairman spoke of the great services which Sir Henry had rendered, not only to this Corporation but to the hospitals throughout the country. Sir Perceval referred to his various activities, particularly his journalistic work and his work as founder of the Royal National Pension Fund for Nurses. It was resolved to record upon the minutes the Committee's sorrow, and their sense of the loss which the Seamen's Hospital Society has suffered.

The House Committee of St. Bartholomew's Hospital, at its meeting on May 13, "received with much regret the news of the death of Sir Henry C. Burdett, a Governor of the hospital since 1903."

### The Royal National Pension Fund for Nurses.

At its monthly meeting, held on May 13, the first since April 8, the Council unanimously passed a resolution of condolence. It expressed its realisation "that the Fund has sustained an irreparable loss by the death of Sir Henry Burdett, who was one of the founders of the Society, and Deputy Chairman from its establishment to the year 1912, when he became a Vice-President." His "intimate knowledge of all matters pertaining to the nursing profession was of the greatest value to his colleagues on the Council, and they much appreciated his help in questions on which he was pre-eminently qualified to advise."

### "A Guy's Man."

The medical and nursing professions have lost a generous friend and an indefatigable worker in the death of Sir Henry Burdett, which occurred on April 29. There can be few alive now who remember him as a fellow student at Guy's in the early seventies. But those who have come after him will readily admit that, although Sir Henry never qualified, he devoted his great talents to the cause of the sick with a zeal that has rarely been equalled even in the ranks of our profession. His death is a great loss to the nation, and we are proud, as we believe he was also, that we can call him a "Guy's man."—*Guy's Hospital Gazette,* May 15, 1920.

### "Medical Press," May 12, 1920.

"The death of Sir Henry Burdett on the 29th ult., in his seventy-fourth year, has removed a remarkable personality from the hospital world. In his special sphere of activity his reputation was world-wide. It brought him into close contact with the Royal Families of this and other countries, as well as with distinguished persons in all grades of social and business life, who encouraged and supported him in his great undertakings, the eventual success of which now remain as memorials of his energy, enterprise, and laudable ambition. The writer became acquainted with him in 1878, while holding a resident appointment at the Seamen's Hospital, Greenwich, where Sir Henry Burdett was the secretary. In these early days his great avidity for work was a marked feature of his life. He seemed always to be planning schemes for improvement in hospital administration. He became the guide, and practically the chief, of his committee, and a natural confidence in himself made responsibilities to rest lightly upon him. Under his guidance the smooth working of the institution was never subject to interruption. There was a firmness in his administration which, however, was never felt. As an official in authority he acquired the confidence of those over whom he ruled; of criticism of him as secretary there was none. In those early days his personality was marked by a natural geniality and friendliness; his buoyancy of spirit was infective. Life with him seemed to be the embodiment of happiness. While vigorous in work, he displayed the same vigour in play—at the times that he allowed himself recreation. Incidentally, he sang vigorously in the hospital chapel at the Sunday morning services; but it was noticeable that his sonorous tones did not correspond with those of the music. Every young resident during Sir Henry Burdett's secretaryship must have come to regard him as a friend with whom it was a pleasure to be associated. The interesting and detailed obituary of its founder which appears in our contemporary, The Hospital, records so fully the many activities of his life work as to constitute the nucleus of a biography. The influence that he was able to wield in the hospital world, the strenuousness of his labours to promote the well-being of these charities, and the success of his efforts to enlist the sympathy and support of the charitable public in their favour, offer an inducement for the publication of a Life of the late Sir Henry Burdett."

